# Multimodal integration of [^18^F]PSMA-1007 PET/CT semiquantitative parameters and clinicopathological data for predicting prostate cancer metastasis

**DOI:** 10.3389/fonc.2025.1640159

**Published:** 2025-10-15

**Authors:** JiaYing Yang, ZhiLong Ma, HaiTong Hao, Jian Chen, ZhiYong Lv, Qian Zhao, YanMei Li

**Affiliations:** ^1^ Nuclear Medicine Department, General Hospital of Ningxia Medical University, Yinchuan, Ningxia, China; ^2^ College of Basic Medical Sciences, Ningxia Medical University, Yinchuan, Ningxia, China; ^3^ Nuclear Medicine Department, International Medical Center Hospital, Xi'an, Shanxi, China; ^4^ Urinary surgery Department,General Hospital of Ningxia Medical University, Yinchuan, Ningxia, China

**Keywords:** [18F]PSMA-1007, positron emission tomography/computed tomography, predictingprostate cancer metastasis, multimodal prediction, machine learning, SHAP

## Abstract

**Background:**

Prostate cancer is one of the most prevalent malignant tumors of the male genitourinary system. The occurrence of metastasis significantly influences treatment strategies and prognosis. However, current risk assessments for metastatic disease primarily rely on single imaging or pathological indicators, which are often limited by suboptimal accuracy and considerable individual variability.

**Objective:**

This study aimed to develop a high-performance predictive model for prostate cancer metastasis by integrating semiquantitative parameters from [^18^F]PSMA-1007 PET/CTwith key clinicopathological features.

**Methods:**

We retrospectively analyzed data from prostate cancer patients, includingPSMA PET/CT-derived features (SUVmax, SUVmean, PSMA-TVp, TL-PSMAp) and clinical-pathological variables (age, tPSA, Gleason score). Five machine learningalgorithms—Logistic Regression, Support Vector Machine, Random Forest, Naive Bayes, and XGBoost—were evaluated for metastasis prediction performance. Model performance was assessed using accuracy, sensitivity, precision, and area under the ROC curve (AUC). Shapley additive explanations (SHAP) were applied to interpret the most effective model.

**Results:**

Among the five algorithms, the XGBoost model achieved an accuracy of 90.32%, sensitivity of 90.0%, specificity of 94.74%, and an AUC of 0.8977. SHAP analysis identified PSMA-TVp, TL-PSMAp as the most important predictors, followed by SUVmax, tPSA, and Gleason score. These findings highlight the key role of PSMA-derived tumor burden in metastasis prediction. Force plots further revealed the individual-level contributions of features, supporting the model’s clinical interpretability.

**Conclusion:**

The XGBoost-based multimodal model integrating PET/CT semiquantitative parameters with clinicopathological data demonstrated excellent accuracy and interpretability in predicting prostate cancer metastasis. This approach has strong potential for clinical application and may provide a valuable tool for personalized treatment decision-making.

## 1 Introduction

Prostate cancer (PCa) is one of the most common malignancies among male worldwide and remains a leading cause of cancer-related death, ranking sixth in global male cancer mortality rates (1). With the growing trend of population aging, the incidence of prostate cancer continues to rise annually, posing a significant challenge to global public health. Clinical studies have shown that patients with metastatic prostate cancer exhibit a markedly reduced 5-year survival rate of approximately 31%, substantially lower than that of patients with localized disease (2). However, the biological behavior of prostate cancer is highly heterogeneous, leading to vastly different progression trajectories and therapeutic responses among patients even at the same clinical stage (2–4). Therefore, the early and accurate identification of patients at high risk of metastasis has become a critical issue in improving treatment outcomes and prolonging survival, and holds great clinical importance for the realization of precision diagnosis and therapy in prostate cancer.

The total prostate-specific antigen (tPSA) can be used for prostate cancer risk stratification and prediction of distant metastasis; however, its specificity is limited, which may lead to unnecessary prostate biopsies in some patients (5). Magnetic Resonance Imaging (MRI) has played a significant role in improving the detection rate and local staging of prostate cancer. Nevertheless, it may still miss approximately 20% of clinically significant cancers and has limited sensitivity and specificity in detecting lymph node metastases (6).

By contrast, Prostate-specific membrane antigen (PSMA), a transmembrane glycoprotein that is highly overexpressed in prostate cancer cells, particularly in advanced or castration-resistant stages. PSMA-targeted PET/CT imaging has demonstrated outstanding sensitivity and specificity in the diagnosis, staging, recurrence detection, and treatment evaluation of prostate cancer (6–10). Compared to conventional imaging techniques, PSMA PET/CT offers significant advantages in detecting small lesions and identifying recurrent disease even in cases with low total prostate-specific antigen (tPSA) levels, thus providing a reliable basis for precision therapy (11, 12). In addition, PSMA PET/CT enables the acquisition of multiple semiquantitative parameters that reflect tumor PSMA expression and volumetric characteristics, such as maximum standardized uptake value (SUVmax), mean standardized uptake value (SUVmean), prostate PSMA-tumor volume (PSMA-TVp) and prostate total lesion PSMA (TL-PSMAp). These quantitative metrics provide an objective basis for evaluating tumor aggressiveness and metastatic potential. Specifically, SUVmax indicates the peak uptake within the most active part of the lesion and is easy to obtain and compare across patients. However, it neglects tumor volume and heterogeneity, potentially underestimating tumor burden.SUVmean reflects the average PSMA ligand uptake across the lesion, offering insight into overall PSMA expression, although it is susceptible to variation depending on the definition of the volume of interest (VOI). PSMA-TVp and TL-PSMAp, as composite indicators of tumor burden, quantify the PSMA-avid tumor volume and the prostate total uptake PSMA, respectively. These parameters offer a more comprehensive assessment of the tumor’s global PSMA ligand uptake and biological behavior and have been reported in numerous studies to be closely associated with tumor staging, metastasis, and prognosis (13–16). However, in current clinical practice, interpretation of these imaging parameters largely relies on empirical assessment, lacking systematic analysis and quantitative predictive methodologies. This limitation hinders their full potential in personalized metastatic risk stratification.

With the rapid advancement of artificial intelligence, particularly machine learning (ML) techniques, there is now a promising opportunity to build personalized risk prediction models by integrating multimodal medical data (17–19).Machine learning algorithms excel in handling high-dimensional, multi-variable, and non-linear data relationships, and have shown great success in early detection, metastasis prediction, and prognosis evaluation across various solid tumors, such as breast and lung cancer (20–23). In the context of prostate cancer, combining machine learning with PSMA PET/CT-derived semiquantitative metrics, clinical features, and pathological data could enable the development of high-performance predictive models, allowing for early identification of high-risk patients and supporting more precise stratified management and clinical decision-making.

In this study, we propose a multimodal data fusion strategy that integrates clinicopathological features (e.g., age, Gleason score) and PSMA PET/CT semi-quantitative metrics (e.g., SUVmax,PSMA-TVp) to construct a machine learning-based predictive model for assessing the risk of metastasis in prostate cancer patients. The goal is to enhance the early identification of high-risk individuals, facilitate personalized treatment planning, and provide an intelligent decision-support tool for risk stratification. Ultimately, this approach aims to shift the paradigm of prostate cancer management from empirical judgment to data-driven precision medicine, with significant clinical value and application potential.

## 2 Materials and methods

### 2.1 Study population

**Figure 1:**
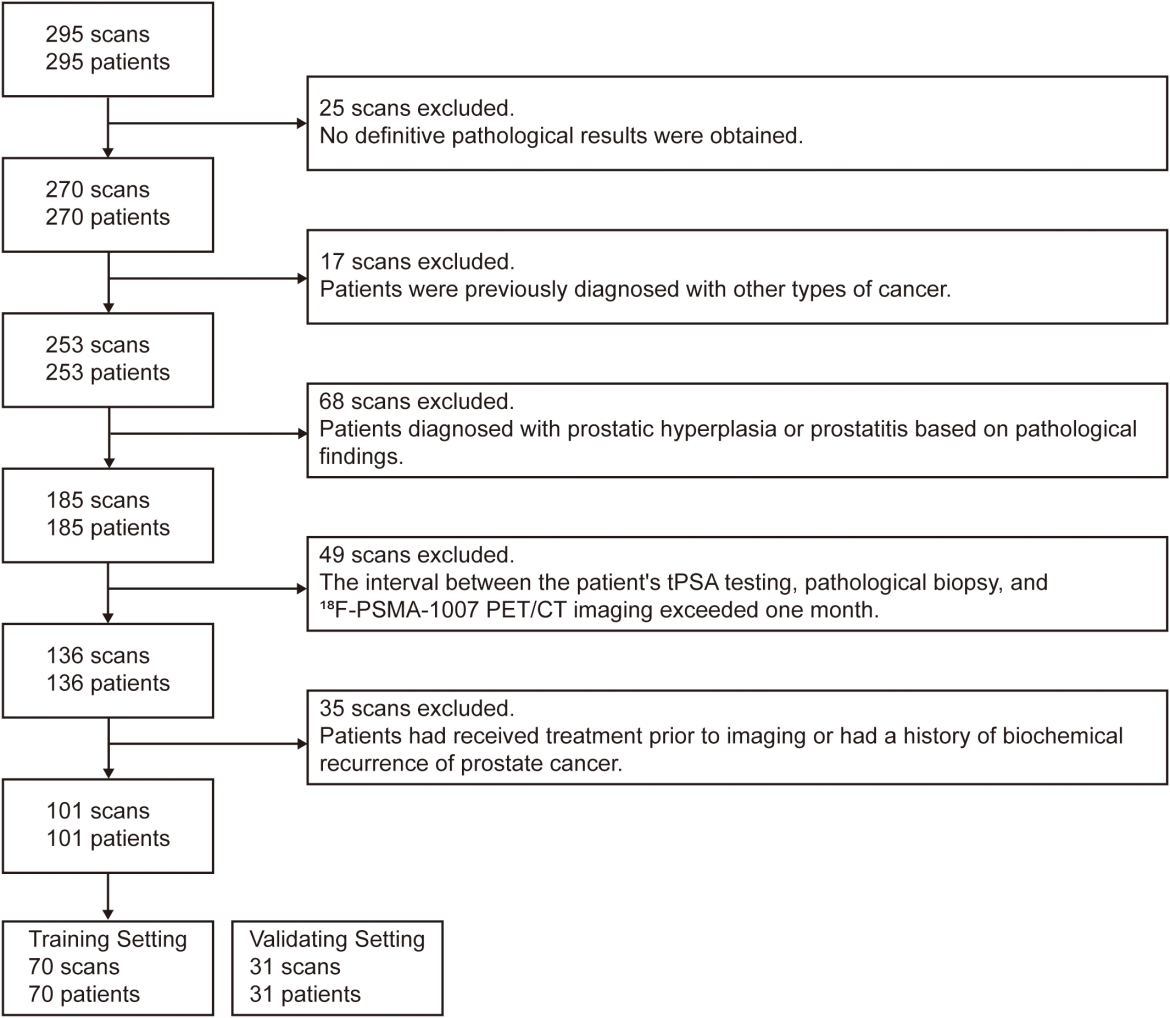
Flowchart illustrating the inclusion and exclusion criteria for patient selection in the study.

This retrospective study included a total of 295 patients with histologically confirmed prostate cancer (PCa) who underwent [^18^F]PSMA-1007 PET/CT imaging at our institution between January 2020 and February 2022. Inclusion criteria were: (1) complete clinical data; (2) patients had undergone transrectal ultrasound-guided prostate biopsy or radical prostatectomy with definitive pathological diagnosis. Exclusion criteria were as follows: (1) presence of other malignancies; (2) an interval of more than one month between serum total prostate-specific antigen (tPSA) testing, pathological biopsy, and [^18^F]PSMA-1007 PET/CT imaging; (3) severe hepatic or renal dysfunction; (4) prior anti-tumor treatment before imaging; (5) lack of PSMA uptake in the primary tumor. After screening, 101 patients met all criteria and were included in the final analysis, the selection process is shown in **Figure 1**.

All study procedures were conducted in accordance with the Declaration of Helsinki and were approved by the hospital ethics committee (Approval Nos. 2020–083 and 2020-876). Written informed consent was obtained from all participants.

### 2.2 Image interpretation

PET/CT imaging was performed using a GE Discovery VCT scanner (64-slice CT), with routine quality control to ensure stable performance. [^18^F]PSMA-1007 was synthesized using the PET-IFB-X5 automated module from Shaanxi Zhengze Biotechnology Co., Ltd., and its radiochemical purity was confirmed by high-performance liquid chromatography to be ≥95%. The injected dose of [^18^F]PSMA-1007 was 4.0 MBq/kg. Whole-body PET/CT scans were performed 60–90 minutes post-injection. Spiral CT scans were first acquired from the skull vertex to mid-thigh with the following parameters: 140 kV tube voltage, 150 mA tube current, 0.875 mm pitch, 3.75 mm slice thickness, and a 512×512 matrix. Subsequently, PET images were acquired over the same range using a 3D acquisition mode with a 128×128 matrix, 2.5 minutes per bed position, and 6–7 bed positions in total. All PET images were attenuation-corrected using the corresponding CT data and reconstructed for image fusion and further analysis.

### 2.3 Image interpretation

All images were independently reviewed in a double-blind manner by two board-certified nuclear medicine physicians with extensive diagnostic experience. Discrepancies were resolved through joint discussion to reach a consensus diagnosis.

On visual inspection, lesions showing focal PSMA uptake higher than the surrounding normal tissue in the prostate were considered positive. A circular region of interest (ROI) was manually drawn on axial images around the lesions, and the positive volume was delineated using a fixed threshold method set at 40% of the SUVmax. The maximum standardized uptake value (SUVmax), the mean standardized uptake value (SUVmean), PSMA-TVp and TL-PSMAp were recorded for each lesion.

Criteria for Lymph Node Metastasis: On [^18^F]PSMA-1007 PET/CT, focal abnormal radiotracer uptake outside of physiological uptake regions (e.g., salivary glands, liver, gallbladder, prostate, kidneys, intestines) was interpreted as metastatic unless located in known false-positive sites such as axillary, mediastinal, or inguinal lymph nodes. The number of metastatic lymph nodes and their corresponding SUVmax, SUVmean, PSMA-TVp and TL-PSMAp were recorded.

Criteria for Bone Metastasis: Focal areas of increased PSMA uptake in bone were considered metastatic if they could not be attributed to fractures, degenerative changes, or other benign bone conditions (24).

Final diagnoses were established based on histopathological findings from surgery or biopsy when available, or through clinical follow-up. For lesions not amenable to tissue diagnosis (e.g., bone or distant metastases), a comprehensive judgment was made based on synchronous imaging findings and clinical follow-up data.

### 2.4 Statistical analysis

All statistical analyses were conducted using Python 3.10. Continuous variables were compared using either Student’s t-test or the Mann–Whitney U test, depending on data distribution. Categorical variables were compared using Pearson’s χ² test or Fisher’s exact test. The dataset was randomly divided into training and validation sets at a ratio of 7:3. For classification model evaluation, receiver operating characteristic (ROC) curves were plotted using probability scores ranging from 0 to 1, and the area under the curve (AUC) was calculated to assess discriminatory performance. A p-value< 0.05 was considered statistically significant.

## 3 Results

### 3.1 Comparison of basic patient information and clinicopathological characteristics

A total of 101 patients with prostate cancer (PCa) were retrospectively enrolled in this study, with a mean age of 68 years. Among them, 97.03% were diagnosed with acinar adenocarcinoma, while the remaining subtypes included one case each of signet ring cell carcinoma, intraductal carcinoma, and foamy gland adenocarcinoma. As summarized in **Table 1**, the Gleason scores ranged from 6 to 10, with 60.39% (61/101) scoring greater than 8. The total prostate-specific antigen (tPSA) levels ranged from 5.42 to 100.0 ng/mL, with 59.41% (60/101) ≥20 ng/mL. Among the cohort, 34 patients showed no evidence of metastasis, while 67 patients had confirmed metastatic disease, including 53 cases of lymph node metastasis, 52 cases of bone metastasis, and 8 cases of visceral metastasis, all of which were pulmonary.

**Table 1:** Baseline feature distribution and dataset partitioning.

Characteristics	Overall cohort N=101	Training setting N=70	Validating setting N=31	*p* value
Age(years)				0.88
Mean±SD	68.91±7.20	68.98±7.27	68.74±7.16	
Gleason (6-10)				0.86
≥8, n(%)	61(60.39)	44(62.86)	17(54.84)	
<8, n(%)	40(39.60)	26(37.14)	14(45.16)	
tPSA (ng/ml)				0.62
≥20, n(%)	60(59.41)	44(62.86)	16(51.61)	
<20, n(%)	41(40.59)	26(37.14)	15(48.39)	
Mean±SD	48.67±46.84	50.27±49.27	45.07±41.36	
Pet parameters				
SUVmax, Mean ± SD	18.79±13.65	17.81±12.76	20.99±15.46	0.29
SUVmean, Mean±SD	11.12±9.00	10.20±7.32	13.15±11.86	0.18
PSMA-TVp, Mean ± SD	17.51±19.97	19.49±21.59	13.06±15.11	0.17
TL-PSMAp, Mean ± SD	171.63±202.69	187.27±218.89	136.32±157.74	0.56
Pathological type, n(%)				0.71
Acinar Adenocarcinoma	98(97.03)	67(95.71)	31(100)	
Others	3(2.97)	3(4.29)	0(0)	
Metastasis, n(%)				
Yes	67(66.34)	47(67.14)	20(64.52)	
No	34(33.66)	23(32.86)	11(35.48)	

**Figure 1** illustrates the distribution of various features between the metastasis and non-metastasis groups. Based on the distributions shown, there are significant differences in several features between the two groups. Specifically, the metastasis group had significantly higher values in tPSA, Gleason score, SUVmax, SUVmean, PSMA-TVp, and TL-PSMAp compared to the non-metastasis group, with all differences being statistically significant (p< 0.05). However, there was no statistically significant difference in age between the two groups (p = 0.096). For details, see **Supplementary Table 1**.

### 3.2 Performance evaluation of machine learning models

To ensure the scientific rigor and effectiveness of model training, the dataset was randomly divided into a training set and a validation set at a 7:3 ratio. The training set included 70 patients, while the validation set included 31 patients. Among the training cohort, 47 patients (67.14%) presented with metastatic disease; in the validation cohort, 20 patients (64.52%) had confirmed metastases. To assess feature distribution balance, we performed homogeneity tests on clinical, pathological, and imaging characteristics between the two cohorts. The results demonstrated no statistically significant differences between the training and validation sets in terms of age, tPSA, Gleason score, SUVmax, SUVmean, PSMA-TVp, and TL-PSMAp (p > 0.05), as shown in **Table 1**. These findings confirm that the two groups were well-balanced across key features, thereby minimizing potential bias introduced by data partitioning during model development and evaluation.

**Figure 2:**
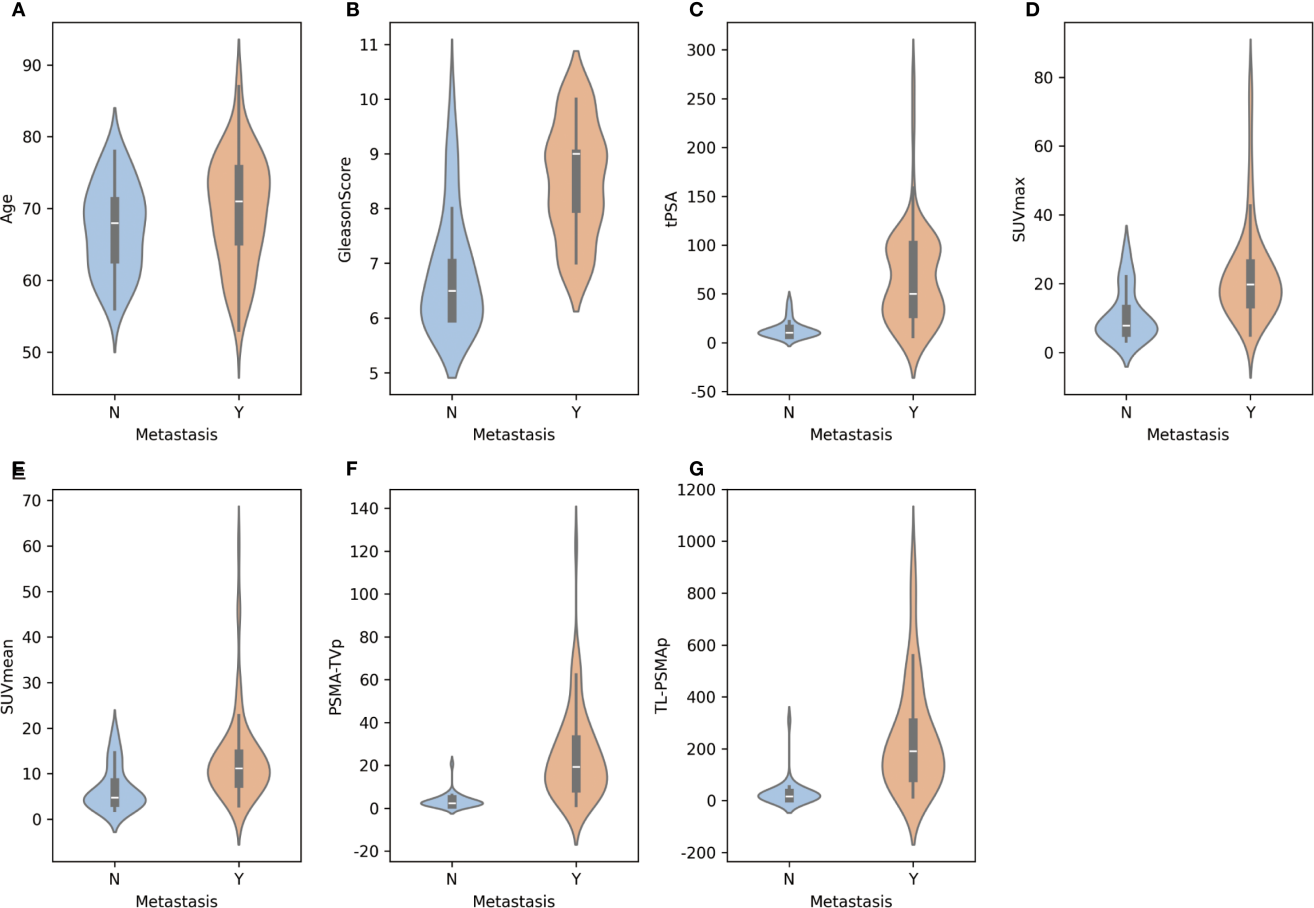
Distribution of individual features between metastatic and non-metastatic prostate cancer patients.**(A-G)** Illustration of the distribution of key features—including age, tPSA, Gleason score, SUVmax, SUVmean, PSMA-TVp, and TL-PSMAp—between metastatic and non-metastatic groups using violin plots. Each subplot shows the kernel density estimation and boxplot for a given feature, allowing visualization of both the value distribution and central tendency. The x-axis represents metastasis status, while the y-axis indicates the corresponding feature values.

In the training cohort, five commonly used classification algorithms—Logistic Regression, Support Vector Machine (SVM), Random Forest, Extreme Gradient Boosting (XGBoost), and Naive Bayes—were employed to construct predictive models. All models were trained using the same set of input features, with the presence or absence of metastasis serving as the output label. In the validation cohort, model performance was comprehensively evaluated using multiple metrics. Receiver Operating Characteristic (ROC) curves were plotted, and the Area Under the Curve (AUC) was calculated to assess classification performance. In addition, accuracy, sensitivity, and specificity were used as complementary evaluation metrics. The ROC curves for each model are shown in **Figure 2A**. To further quantify the overall performance of each model across multiple metrics, a Composite Score was introduced, calculated as follows: Composite_Score= 0.4×AUC+0.3×F1_score+0.1× Accuracy+0.1×Sensitivity+0.1×Specificity . This score provides a balanced and representative evaluation by integrating both discriminative power and classification effectiveness. **Figure 2B** illustrates the radar chart of the six major performance indicators, clearly highlighting the superior overall performance of the XGBoost model.

Furthermore, to analyze the types and patterns of classification errors, confusion matrices of the five models in the validation cohort were generated (**Figures 2C–G**). Among them, the XGBoost model demonstrated the best performance in predicting prostate cancer metastasis, achieving an AUC of 0.8977, an accuracy of 90.32%, a sensitivity of 90.0%, and a specificity of 94.74% in the validation set. The Naive Bayes model ranked second, with the same AUC (0.8977) and a slightly higher sensitivity (95.0%), though its accuracy (87.10%) and specificity (86.36%) were slightly lower. In contrast, the Logistic Regression, Support Vector Machine, and Random Forest models showed relatively inferior performance across the evaluated metrics.

In summary, all models demonstrated a certain degree of discriminative ability in the validation set; however, nonlinear ensemble models such as XGBoost exhibited superior generalization and robustness when integrating multiple features. Combined with the performance visualizations in **Figure 2**, these results suggest that deep ensemble learning methods hold greater potential for clinical application in predicting prostate cancer metastasis risk.

### 3.3 Feature contribution and interpretability of the XGBoost model

Based on the performance evaluation of all models, we ultimately selected the XGBoost model for predicting prostate cancer metastasis, as it demonstrated the best overall performance. To further explore the model’s decision-making process and the contribution of each input feature, we employed the SHAP method for interpretability analysis. SHAP is a game-theory-based model interpretation technique that assigns each feature a clear “contribution value,” quantifying both the direction and magnitude of its impact on model output. Compared to traditional feature importance analysis, SHAP not only reflects the global importance of features but also supports fine-grained explanations at the individual level. In this study, we used SHAP to interpret the XGBoost model from both global and individual perspectives.

**Figure 3:**
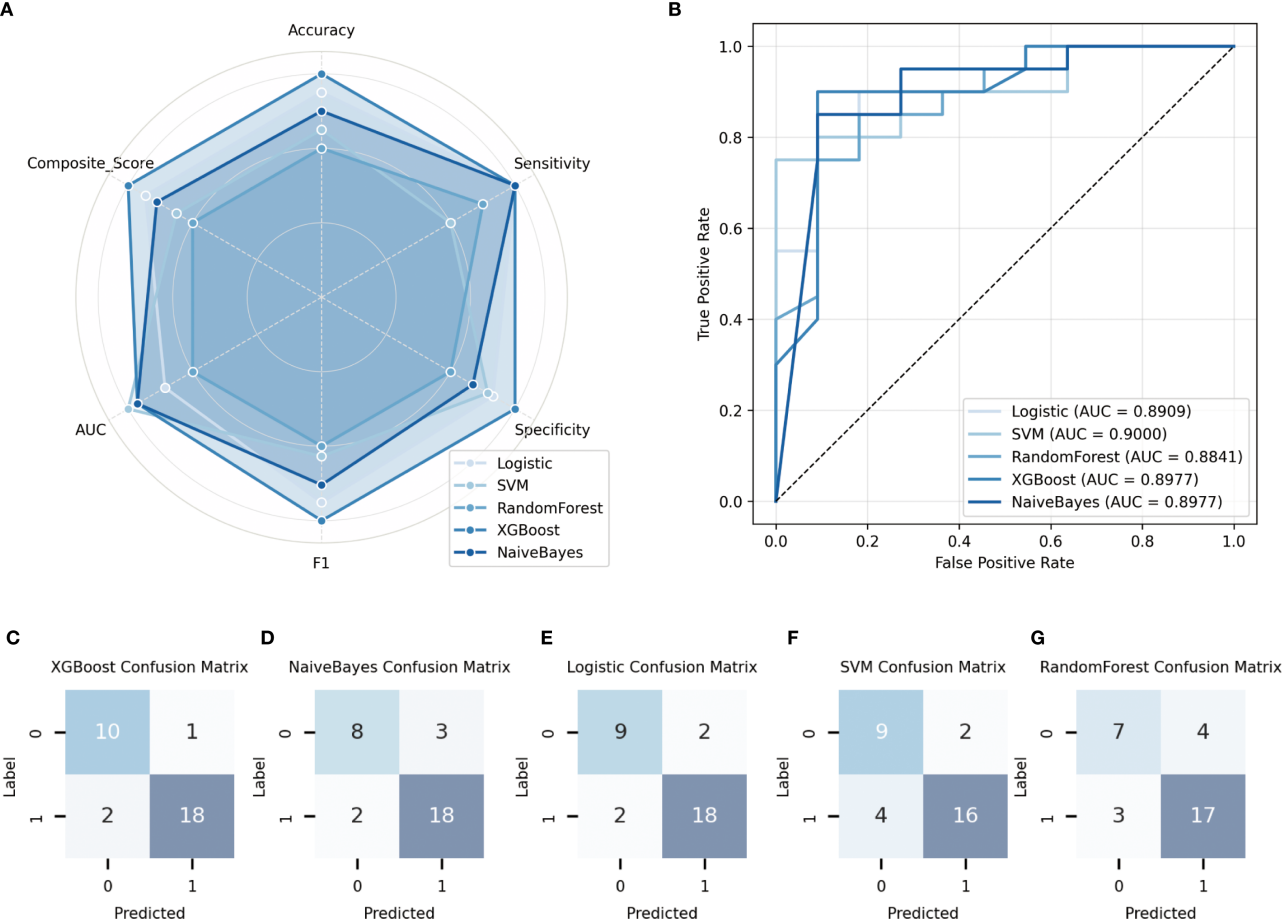
Performance evaluation of different models on the validation set. **(A)** Receiver operating characteristic (ROC) curves of the five models, along with their corresponding area under the curve (AUC) values; **(B)** Radar plots illustrating the comparative performance of five classification algorithms in terms of accuracy, sensitivity, specificity, and other key metrics; **(C–G)** Confusion matrices depicting the classification results of each model on the validation set.

As shown in **Figure 3**, subplot A presents the SHAP summary plot for the XGBoost model, illustrating the global feature importance rankings and their influence on metastasis prediction. The horizontal axis represents SHAP values (i.e., the impact on the prediction), while the vertical axis lists the eight input features. Each dot represents a sample’s SHAP value for that feature, with color gradients from blue to red corresponding from low to high feature values.

The SHAP analysis revealed that PSMA-TVp, and TL-PSMAp, as key PET parameters reflecting the overall tumor burden based on PSMA expression, contributed the most to the prediction of prostate cancer metastasis. This suggests that the tumor’s overall PSMA expression holds significant predictive value for metastasis risk. SUVmax, representing the highest uptake intensity of the most active part of the lesion, showed a slightly lower contribution due to its sensitivity to image noise and lesion heterogeneity, compared to the more comprehensive PSMA-TVp, and TL-PSMAp. Traditional clinical indicators such as tPSA and Gleason score also demonstrated strong discriminative power in the model, indicating that fundamental serological and histopathological features still play a stable role in prediction. Although SUVmean can reflect the overall tumor PSMA expression of the lesion, its importance was slightly lower due to its sensitivity to VOI (volume of interest) delineation. Age had the least predictive contribution in the model, consistent with the lack of statistical difference between groups, suggesting its limited value in distinguishing metastasis risk within this cohort.

**Figure 4:**
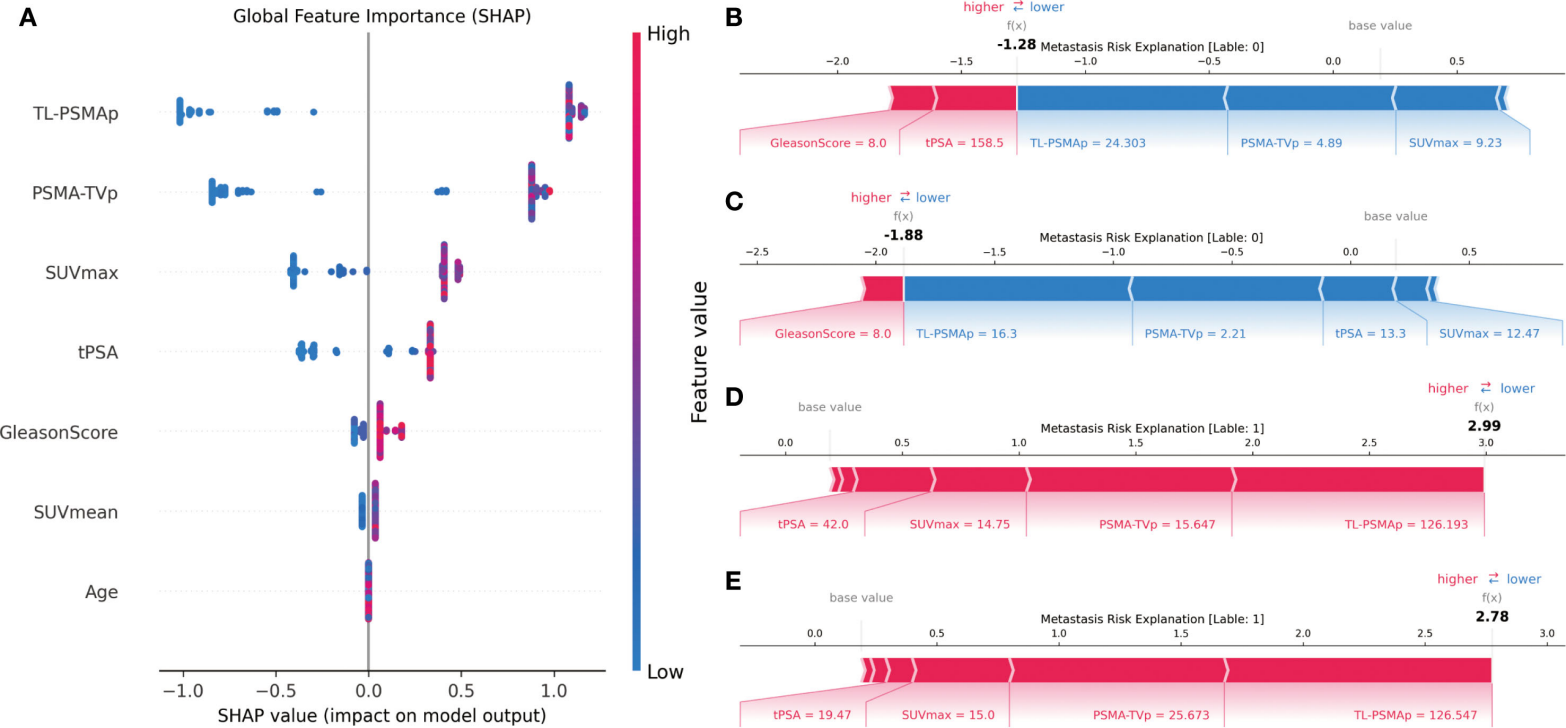
SHAP-based interpretability analysis of the XGBoost model. **(A)** SHAP summary plot illustrating the global importance and directional impact of each feature on the model’s prediction; **(B, C)** SHAP force plots for two non-metastatic patients, showing how low metabolic values drive predictions toward the “non-metastatic” class; **(D, E)** SHAP force plots for two metastatic patients, where higher SUVmax,PSMA-TVpand TL-PSMAp values strongly contribute to the model’s prediction of metastasis.

To further illustrate the model’s decision-making mechanism at the individual level, this study randomly selected two non-metastatic patients and two metastatic patients, and generated SHAP force plots for each case (**Figure 4**, subplots B–E). Subplots B and C correspond to the non-metastatic patients. As shown in the plots, features such as PSMA-TVp, and TL-PSMAp, and SUVmax were all at relatively low levels, contributing negatively to the prediction outcome and steering the model toward a “non-metastatic” classification. Although tPSA in subplot B and the Gleason score in subplot C showed some positive influence on the prediction, the overall SHAP value contributions still supported a “non-metastatic” result. Subplots D and E illustrate the SHAP explanations for the two metastatic patients. In these cases, PSMA-TVp, TL-PSMAp, SUVmax, and tPSA were markedly elevated, exhibiting strong red positive forces that drove the model decisively toward a “metastatic” prediction. These results indicate that features reflecting high PSMA expression and tumor burden play a critical role in the model’s decision-making process, further confirming their clinical potential in identifying the risk of prostate cancer metastasis.

These individual-level explanations demonstrate that the model not only possesses strong overall predictive performance but also clearly reveals the key features and their directional influence at the level of individual patients. This enhances the interpretability and clinical applicability of the model. Combined with the SHAP analysis results, it is evident that PSMA PET/CT-derived parameters such as PSMA-TVp and TL-PSMAp play a dominant role in predicting prostate cancer metastasis, outperforming the traditional SUVmax metric. Meanwhile, clinicopathological variables such as the Gleason score and tPSA also show significant predictive value, suggesting that imaging biomarkers and pathological indicators offer complementary strengths in this task.

In summary, the SHAP-based interpretability analysis not only confirmed the critical role of PSMA-avid tumor burden-related parameters in predicting prostate cancer metastasis but also highlighted the potential of the XGBoost model in providing individualized risk assessments. This approach holds promise for supporting data-driven clinical decision-making and guiding stratified management and treatment strategies for prostate cancer.

## 4 Discussion

The occurrence of prostate cancer metastasis is directly related to treatment decisions and prognosis assessment. To enhance clinical prediction capabilities, this study developed a multimodal metastasis risk prediction model based on machine learning by integrating semiquantitative parameters from PSMA PET/CT, clinical variables, and pathological features. This comprehensive approach provides a powerful tool for precision diagnosis and treatment of prostate cancer. The proposed model demonstrated excellent performance in the validation cohort, achieving an accuracy of 90.32%, sensitivity of 90.0%, specificity of 94.74%, and an area under the curve (AUC) of 0.8977. These metrics indicate strong discriminative ability and suggest that the model can effectively support clinicians in identifying patients at high risk of metastasis. Previous studies have predominantly focused on lesion-level prediction using PSMA PET/CT imaging features or on evaluating treatment response following radioligand therapy (25–27). In contrast, this study transcends the conventional paradigm of single-modality prediction by systematically integrating heterogeneous data sources.According to the D’Amico risk classification, metastasis can be observed in at least half of patients categorized as high risk, underscoring its clinical relevance. Nevertheless, this system does not incorporate molecular imaging information (28, 29). By leveraging the complementary use of multidimensional features, the model’s predictive performance was substantially improved. This multimodal fusion approach not only enhances risk stratification accuracy but also offers a novel technical pathway for intelligent prediction of metastatic PCa, further expanding the application scope of machine learning in prostate cancer management.

As an emerging technology, machine learning is still in its early stages of clinical application but has already demonstrated broad potential in biomedical research. The predictive model developed in this study provides strong supporting evidence for the clinical translation of machine learning methods in urology. XGBoost, a type of ensemble learning algorithm, has shown superior performance in various medical prediction tasks due to its powerful nonlinear modeling capabilities and adaptability to high-dimensional, heterogeneous data (30–32). In our study, XGBoost outperformed other models such as Random Forest and Support Vector Machine in assessing the risk of prostate cancer metastasis. Unlike traditional models that rely on a single imaging or clinical-pathological feature, our approach integrates imaging, clinical, and pathological data to enhance the model’s ability to identify complex patterns of metastasis. This multi-source data integration strategy allows for a more comprehensive representation of tumor biology and individual patient characteristics, and its effectiveness has also been demonstrated in fields such as head and neck cancer and cardiovascular disease (33, 34).

The SHAP framework, as a leading tool for model interpretability, effectively unveils the “black-box” mechanisms within machine learning models. In this study, SHAP analysis of the XGBoost model’s decision-making process revealed that PSMA-TVp, and TL-PSMAp made the largest marginal contributions, suggesting that volumetric parameters exhibit greater stability and discriminative power in predicting prostate cancer metastasis. Compared to SUVmax and SUVmean, which only reflects the highest uptake within a single voxel, PSMA-TVp, and TL-PSMAp integrate both PSMA expression level and lesion volume, offering a more comprehensive representation of tumor burden. This allows them to demonstrate superior discriminative capacity and robustness under complex biological conditions. These findings are consistent with previous studies and further validate their potential clinical value in capturing tumor heterogeneity and identifying distant metastasis (35, 36). Although SUVmax, a conventional PSMA PET/CT parameter, retained some importance in the model, its interpretive capacity was limited due to its reflection of only the local peak uptake, making it susceptible to noise interference (37, 38). Clinical and pathological variables such as tPSA and Gleason score also contributed significantly to the prediction task, indicating that fundamental serological and histological grading information provides important complementary value to the mode (39, 40). In contrast, age showed the lowest contribution, which aligns with its lack of statistical difference between groups, suggesting its limited predictive value for metastasis risk within this study cohort. Overall, the XGBoost model, when applied to high-dimensional multimodal data, tends to prioritize variables with stable global explanatory power. This highlights the importance of incorporating volumetric PSMA-avid tumor parameters to enhance model performance. Future research should consider giving priority to such comprehensive indicators in clinical applications to improve model generalizability and decision-making utility. Overall, the XGBoost model, when applied to high-dimensional multimodal data, tends to prioritize variables with stable global explanatory power. This highlights the importance of incorporating volumetric PSMA-avid tumor parameters to enhance model performance. Future research should consider giving priority to such comprehensive indicators in clinical applications to improve model generalizability and decision-making utility.

This study has several limitations. First, it is a single-center retrospective analysis with a relatively limited sample size(n=101), although well-balanced across clinical variables, may limit the generalizability of our findings to broader and more heterogeneous patient populations. Larger prospective, multicenter studies are warranted to validate and refine the predictive performance of our model in diverse clinical settings. Second, although SHAP analysis was employed to enhance model interpretability, the misclassification mechanisms for borderline cases require further investigation. Third, the current model is primarily based on structured features. Future studies may consider integrating raw imaging data, genomic information, longitudinal dynamic indicators, and additional clinical risk stratification systems such as the D’Amico classification to further enhance predictive accuracy and robustness.Additionally, the clinical deployment and user interaction workflows of the model remain to be designed and optimized to ensure feasibility and usability in real-world medical settings.

## 5 Conclusions

In conclusion, the XGBoost model accurately predicted prostate cancer metastasis, with PET parameters PSMA-TVp, TL-PSMAp, and SUVmax contributing more prominently than traditional clinical indicators such as Gleason score and tPSA.

## Data Availability

The original contributions presented in the study are included in the article/**Supplementary Material**. Further inquiries can be directed to the corresponding authors.
