# Supplementary material for: Multimodal integration of [18F]PSMA-1007 PET/CT semiquantitative parameters and clinicopathological data for predicting prostate cancer metastasis
Source: Front Oncol. 2025 Oct 15;15:1640159. doi: 10.3389/fonc.2025.1640159 (PMC12568342; doi:10.3389/fonc.2025.1640159)
Supplement: Supplementary file 1 [file Table1.docx]

**Sup Table 1 | Comparison of Basic Patient Information and Clinical** **Characteristics**

| Characteristics | Metastasis  (N=67) | Non-Metastasis  (N=34) | *p* value |
| --- | --- | --- | --- |
| Age(years) |  |  | 0.096 |
| Mean±SD | 69.76±7.61 | 67.23±6.09 |  |
| Gleason (6-10) |  |  | 1.93e-9 |
| ≥8, n(%) | 54(80.60) | 7(20.59) |  |
| ＜8, n(%) | 13(19.40) | 27(79.41) |  |
| tPSA (ng/ml) |  |  | 8.48e-12 |
| ≥20, n(%) | 55(82.09) | 5(14.71) |  |
| ＜20, n(%) | 12(17.91) | 29(85.29) |  |
| Mean±SD | 66.36±48.38 | 13.83±8.96 |  |
| Pet parameters |  |  |  |
| SUVmax, Mean ± SD | 22.81±14.33 | 1086±7.47 | 1.35e-7 |
| SUVmean, Mean±SD | 13.32±9.83 | 6.74±4.77 | 2.47e-6 |
| PSMA-TVp, Mean ± SD | 24.63±21.11 | 3.50±3.47 | 4.96e-13 |
| TL-PSMAp, Mean ± SD | 243.79±212.59 | 29.44±52.06 | 3.43e-13 |
| Pathological type, n(%) |  |  | 0.213 |
| Acinar Adenocarcinoma | 66(98.51) | 32(94.12) |  |
| Others | 1(1.49) | 2(5.88) |  |
